# Crosstalk between ABO and Forssman (FORS) blood group systems: FORS1 antigen synthesis by *ABO* gene-encoded glycosyltransferases

**DOI:** 10.1038/srep41632

**Published:** 2017-01-30

**Authors:** Miyako Yamamoto, Emili Cid, Fumiichiro Yamamoto

**Affiliations:** 1 Laboratory of Immunohematology and Glycobiology, Josep Carreras Leukaemia Research Institute (IJC), Campus Can Ruti, Badalona, Barcelona, Spain; 2 Programa de Medicina Predictiva i Personalitzada del Càncer (PMPPC), Institut d′Investigació Germans Trias i Pujol (IGTP), Campus Can Ruti, Badalona, Barcelona, Spain

## Abstract

*A* and *B* alleles at the *ABO* genetic locus specify A and B glycosyltransferases that catalyze the biosynthesis of A and B oligosaccharide antigens, respectively, of blood group ABO system which is important in transfusion and transplantation medicine. *GBGT1* gene encodes Forssman glycolipid synthase (FS), another glycosyltransferase that produces Forssman antigen (FORS1). Humans are considered to be Forssman antigen-negative species without functional FS. However, rare individuals exhibiting A_pae_ phenotype carry a dominant active *GBGT1* gene and express Forssman antigen on RBCs. Accordingly, FORS system was recognized as the 31st blood group system. Mouse *ABO* gene encodes a *cis*-AB transferase capable of producing both A and B antigens. This murine enzyme contains the same GlyGlyAla tripeptide sequence as FSs at the position important for the determination of sugar specificity. We, therefore, transfected the expression construct into appropriate recipient cells and examined whether mouse *cis*-AB transferase may also exhibit FS activity. The result was positive, confirming the crosstalk between the ABO and FORS systems. Further experiments have revealed that the introduction of this tripeptide sequence to human A transferase conferred some, although weak, FS activity, suggesting that it is also involved in the recognition/binding of acceptor substrates, in addition to donor nucleotide-sugars.

The ABO system is one of the most important blood group systems in transfusion medicine^1^,^2^. This system is composed of A and B oligosaccharide antigens expressed on red blood cells (RBCs), and anti-A and anti-B antibodies against those antigens in serum. Although both antigens and antibodies are involved, this hereditary trait is specified by a single genetic locus of *ABO*. Functional *A* and *B* alleles encode blood group A and B glycosyltransferases with similar, but distinct, specificities. A transferase transfers an *N*-acetyl-D-galactosamine (GalNAc) to oligosaccharide acceptor substrate, H substance (Fucα1-2Gal-), to produce A antigen, whereas B transferase transfers a galactose to the same acceptor to produce B antigen. The immunodominant structures of A and B antigens are GalNAcα1-3(Fucα1-2)Gal- and Galα1-3(Fucα1-2)Gal-, respectively. *O* allele-encoded proteins are enzymatically inactive and do not possess either of the transferase activities. Accordingly, H substance remains without further modifications in blood group O individuals. Presence or absence of the antibodies is determined secondarily to the absence or presence of the corresponding antigens. In other words, individuals expressing A and/or B antigens do not possess the antibodies against those antigens, however, individuals not expressing A and/or B antigens possess the antibodies against those antigens (Landsteiner’s Law)^3^,^4^. We previously elucidated the molecular genetic basis of ABO system^5^. *A* and *B* alleles encode proteins that are different by 4 amino acid residues at codons 176, 235, 266, and 268. They are Arg, Gly, Leu, and Gly in A transferase, and Gly, Ser, Met, and Ala in B transferase. The latter 2 codons of the 4 amino acid substitutions determine sugar specificities of A and B transferases, whereas the first and second codons exhibit no and slight effects, respectively. Many *O* alleles are inactive due to a single nucleotide deletion (261delG)^5^, although inactivating missense mutations can also be found (Gly268Arg, for instance)^6^.

The FORS system is another system of RBC polymorphism expressing heterophilic Forssman antigen (FORS1) at low-prevalence. Forssman antigen was initially noted by Frouin and re-discovered by Forssman when antibodies produced by injecting rabbits with a suspension of kidney tissue from guinea pig were found to lyse sheep erythrocytes in the presence of the complement proteins^7^. Those antibodies were later used to classify animals into Forssman antigen-positive and Forssman antigen-negative species, depending on the antigen expression^8^,^9^. For instance, guinea pig, sheep, and mouse are Forssman-positive, whereas rabbit and human are Forssman-negative. Chemical determination showed that Forssman antigen is carried on a pentasaccharide glycolipid with the structure of GalNAcα1-3GalNAcβ1-3Galα1-4Galβ1-4Glcβ1-Cer^10^. Forssman glycolipid synthase (FS) catalyzes the final step of its biosynthesis^11^,^12^. Canine *GBGT1* gene cDNA encoding FS was cloned by Haslam and Baenziger, and *GBGT1* and *ABO* genes were found to be paralogous genes that were derived from the same ancestral gene by gene duplication and subsequent divergence^13^.

Forssman antigen has only lately been taken into consideration for blood transfusion. It was known before that RBCs from rare individuals exhibiting the phenotype named A_pae_ reacted strongly with *Helix pomatia* lectin, weakly with polyclonal anti-A antibodies, but not with monoclonal anti-A antibodies^14^. Because of reactivity to polyclonal anti-A antibodies, A_pae_ was thought to be an A subgroup. However, recent chemical characterization of glycolipids on A_pae_ RBCs has identified Forssman glycolipid^15^,^16^. The molecular genetic analysis demonstrated that A_pae_ individuals had a *GBGT1* gene containing the Arg296Gln substitution when compared with FS protein of ordinary non-A_pae_ individuals. Forssman glycolipid is immunologically and chemically distinct from blood group A and B structures, *GBGT1* gene is different from *ABO* gene, and anti-Forssman antigen antibodies exist. Therefore, the International Society of Blood Transfusion (ISBT) has recognized FORS system as the 31st blood group system with one low-prevalence allele expressing Forssman antigen (FORS1).

Excluding special case of A_pae_ individuals, humans are thought to be Forssman antigen-negative species. Strangely, however, Forssman antigen has been detected in a variety of human tumors, including metastatic tumor of biliary adenocarcinoma in liver^17^, stomach and colon tumors^18^,^19^,^20^, and lung tumors^21^. Forssman antigen expression has also been observed in human cancer cell lines of lung, breast, and stomach^22^,^23^. Why and how Forssman glycan is expressed in cancer remains a mystery.

Over the last 2 decades, we have been investigating *ABO* genes in other species than humans. We first studied primate *ABO* genes, and found that the latter 2 of the 4 amino acid substitutions between human A and B transferases are conserved in primates, depending on A or B specificity^24^. We then cloned porcine A transferase cDNA and demonstrated that the major portion of A transferase coding sequence is missing in the *O* allele of pig AO system^25^. We also cloned *ABO* gene cDNAs from mouse species, and demonstrated that mouse *ABO* gene encodes *cis*-AB transferase, an enzyme that is capable of producing both A and B antigens^26^. In recent years, we have also expanded our studies to the other members of α1,3-Gal(NAc) transferase family of genes. In addition to *GBGT1*, those genes include *GGTA1* genes, which encode α1,3-galactosyltransferases to synthesize α1,3-galactosyl epitope (Galα1-3Galβ1-4GlcNAcβ-), *A3GALT2* genes, which encode isogloboside b3 synthase to synthesize isogloboside b3 (Galα1-3Galβ1-4Glcβ1-Cer), and *GLT6D1* (glycosyltransferase 6 domain containing 1) genes, whose transferase activity remains to be examined.

The gene we initially investigated was *GBGT1*. We compared amino acid sequences of FS proteins between Forssman-positive and Forssman-negative species. We then prepared structural chimeras between functional mouse FS and non-functional human FS protein, determined FS activity by DNA transfection of the expression constructs into COS1 African green monkey kidney cells followed by immunostaining with anti-Forssman monoclonal antibody^27^. We narrowed down the regions responsible for the human inactivity. We then prepared amino acid substitution constructs by *in vitro* mutagenesis, and performed additional DNA transfection-immunostaining experiments. We found that two substitutions (c.688G > A [p.Gly230Ser] and c.887A > G [p.Gln296Arg]) are responsible for the inactivation of human FS. Codon 230 of human/mouse FS proteins corresponds to codon 235 of human A/B transferase, and the glycine residue at this position was previously shown to be important for GalNAc specificity of A transferase^28^. We also demonstrated that the reversion of one of the two substitutions in human FS protein conferred weak FS activity whereas the reversion of the two restored FS activity to a level equivalent of mouse FS. In conclusion, we demonstrated the molecular mechanism that resulted in Forssman negativity in humans. In the same paper we also characterized probable mechanisms of inactivation in several other Forssman-negative species including cow, rat, and rabbit^27^. We also investigated the evolution of α1,3-Gal(NAc) transferase family of genes and produced a phylogenetic tree of those glycosyltransferase genes^29^. One important finding from that study was that the tripeptide sequence corresponding to codons 266–268 of human A and B transferases is conserved to be GlyGlyAla in *GBGT1* genes from all the species that were examined except for cow, 3 Old World monkeys, and several fish species, suggesting that this tripeptide sequence is important for FS activity. Surprisingly, however, the same GlyGlyAla tripeptide was also found in mouse *ABO* gene-encoded *cis*-AB transferase. We, therefore, examined whether or not mouse *cis*-AB transferase may also possess FS activity, in addition to A and B transferase activities.

## Results

### Conservation of GlyGlyAla (GGA) tripeptide sequence in the majority of *GBGT1* gene at the position corresponding to codons 266–268 of human blood group A and B transferases

Among 69 Ensembl species, 46 species were found to possess *GBGT1* gene(s), whereas the remaining 23 have none. Excluding fishes where variations are frequent, *GBGT1* genes from the majority of other species contain GlyGlyAla (GGA) tripeptide sequence at the equivalent position of codons 266–268 of human A and B transferases (Supplemental Table 1). The exceptions are cow containing GlyArgAla (GRA) and three Old World monkeys (macaque, olive baboon, and Vervet Monkey or African Green Monkey (vervet-AGM)) containing GlyGlyLys (GGK). There is a deletion of 33 nucleotides (11 amino acids) at 12 nucleotides (4 amino acids) downstream of the lysine residue in the bovine *GBGT1* gene. Because this region of protein is critical for the catalytic activity of FS proteins, the deletion seems to render cow FS protein enzymatically non-functional. *GBGT1* genes from the 3 primates are unlikely to be functional, either, considering that the substitution of codon 268 of human A and B transferases by the lysine residue abolished either of the transferase activities^30^. It should also be noted that several fish *GBGT1* genes also contain the GlyGlyAla tripeptide. Taken together, it was hypothesized that the conserved GlyGlyAla tripeptide sequence plays a significant role for *GBGT1* gene-encoded FS activity.

### Presence of GlyGlyAla (GGA) tripeptide sequence in the corresponding position of mouse *cis*-AB transferase

**Figure 1:**
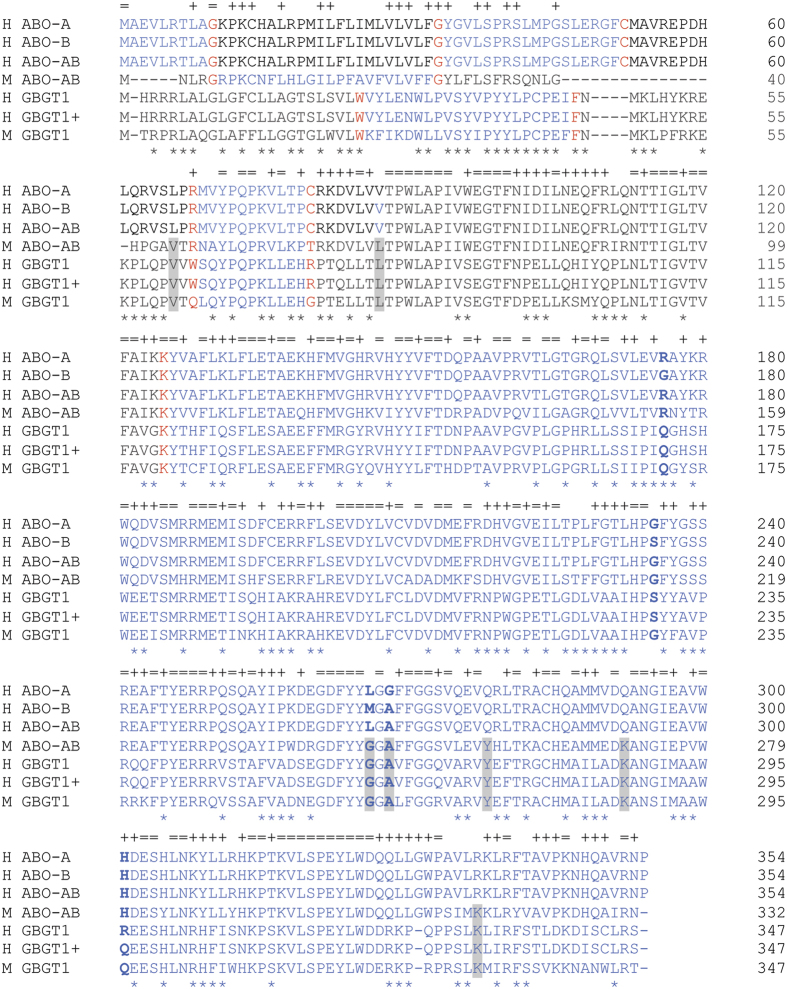
Amino acid sequence comparison between human A/B/*cis*-AB transferases, mouse *cis*-AB transferase, and human and mouse FS proteins. Amino acid sequences of the coding regions of human A, B, and *cis*-AB transferases encoded by *A101* (H_ABO-A), *B101* (H_ABO-B), and *cis-AB01* (H_ABO-AB) alleles of *ABO* gene, mouse *cis*-AB transferase encoded by mouse *ABO* gene (M_ABO-AB), human FS protein encoded by Forssman-negative *GBGT1* gene (H_GBGT1) from ordinary individuals and that of Forssman-positive *GBGT1* gene (H_GBGT1+) from rare A_pae_ individuals, and mouse FS encoded by *GBGT1* gene (M_GBGT1) were aligned and are shown. When amino acids are conserved in all the proteins, they are marked above with an equal symbol (=). 116 out of 274 amino acids (42.3%) in the last 2 coding exons are conserved in all those proteins. When amino acids of mouse *cis*-AB transferase are identical to all the other human A/B/*cis*-AB transferases, they are marked above with a plus symbol (+). Similarly, when amino acids are conserved among FS proteins, they are marked below with an asterisk (*). The seven amino acids that are shared between mouse *cis*-AB transferase and all FS proteins are highlighted. Crucial amino acids corresponding to codons 176, 235, 266, and 268 of human A/B/*cis*-AB transferases, as well as codon 296 of FS proteins are indicated in bold type. Murine *cis*-AB transferase has the same tripeptide sequence of GlyGlyAla(GGA) as that of FS proteins.

The amino acid sequences of blood group A/B/*cis*-AB transferases and FS proteins were MEGA-aligned, and are shown in Fig. 1. The proteins analyzed are human A transferase (H_ABO-A), human B transferase (H_ABO-B), human *cis*-AB transferase (H_ABO-AB), mouse *cis*-AB transferase (M_ABO-AB), human *GBGT1*-encoded non-functional FS protein (H_GBGT1), human *GBGT1* FS with Arg296Gln substitution responsible for A_pae_ phenotype (H_GBGT1 + ), and mouse *GBGT1* FS (M_GBGT1). The amino acids corresponding to codons 176, 235, 266, and 268 of human A and B transferases, as well as codon 296 of FS proteins, are shown in bold type. Those amino acids that are conserved in all the FS proteins and also in mouse *cis*-AB transferase (M_ABO-AB) are highlighted.

Evidently, mouse *cis*-AB transferase is more homologous to human A/B/*cis*-AB transferases than mouse and human FS proteins. Excluding the N-terminal sequences where alignment was difficult, there are only 7 amino acids that are uniquely conserved between mouse *cis*-AB transferase and mouse/human FS proteins, with two situated at codons 245 and 247 of mouse *cis*-AB transferase, which correspond to codons 266 and 268 of human A/B transferases. The tripeptide sequence of GlyGlyAla (GGA) at codons 245–247 of mouse *cis*-AB transferase is identical to the corresponding sequence at codons 261–263 of FS proteins. It should be noted that human *cis*-AB transferase coded by *cis-AB01* allele possesses LeuGlyAla (LGA) and not GlyGlyAla (GGA).

### Enhanced sensitivity of FS activity detection using COS1(B3GALNT1) cells as demonstrated by the re-examination of inactivating mutations in human *GBGT1* gene

**Figure 2:**
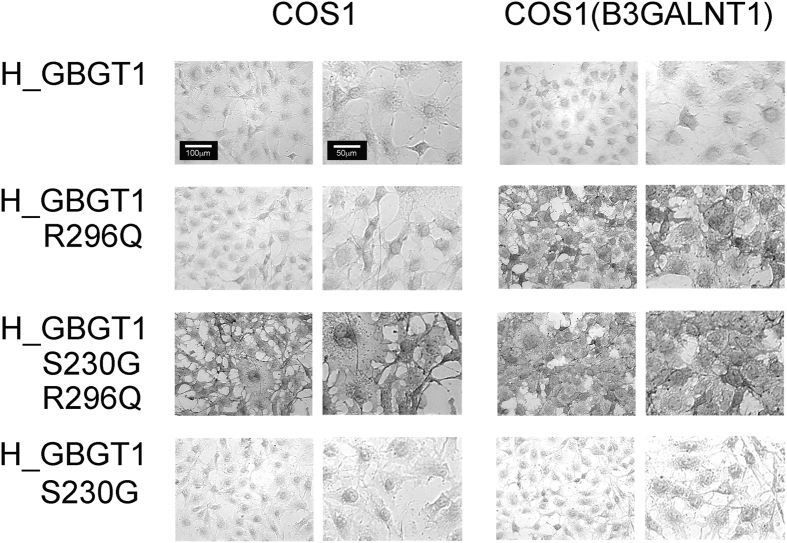
Immunostaining of COS1 and COS1(B3GALNT1) cells using anti-Forssman antibody after infection with viral constructs expressing human FS proteins with and without amino acid substitutions. Results of anti-Forssman antibody immunostaining after viral infection are compared between COS1 and COS1(B3GALNT1) cells. Cells were imaged using a Leica DMI6000B microscope equipped with a 20x/0.30 PH1 objective lens. One representative optical field was selected from individual experiments, and the cell pictures were taken in grey scale at the same magnification using monochrome DFC420 camera and Application Suite V3 software. All pictures are shown in 2 different magnifications. The right pictures correspond to the right bottom quarter of the left pictures at a higher magnification (2x). Figures were created using Adobe Photoshop software. The left panel shows the results using COS1 cells, and the right panel shows those using COS1(B3GALNT1) cells. The viral expression constructs examined are original human *GBGT1* gene (H_GBGT1), human *GBGT1* with Arg296Gln substitution (H_GBGT1-R296Q), human *GBGT1* with Ser230Gly and Arg296Gln substitutions (H_GBGT1-S230G&R296Q), and human *GBGT1* with Ser230Gly substitution (H_GBGT1-S230G) from top to bottom. Because the cells infected with the viral vectors were FACS-selected for their expression of coupled BFP and GFP, all the cells in a single experiment exhibited similar immunostaining results; positive or negative, depending on the constructs. Forssman antigen positivity is characterized not only by darker grey but also by cell surface DAB pigmentation.

We compared the immunostaining results obtained using COS1 cells and those obtained using COS1(B3GALNT1) cells. Four expression constructs of FS proteins were analyzed. Results are shown in Fig. 2. Using COS1 cells, only the double revertant H_GBGT1(S230G&R296Q) construct exhibited FS activity. However, by using COS1(B3GALNT1) cells, not only H_GBGT1(S230G&R296Q) construct, but also H_GBGT1(R296Q) construct exhibited FS activity, suggesting that enhanced availability of globoside, the enzymatic reaction product of *B3GALNT1* gene-encoded β1,3-*N*-acetyl-galactosaminyltransferase 1 and the acceptor substrate for FS, complemented the deficiency due to the Gly230Ser substitution in human *GBGT1* gene. In other words, under the condition where a higher concentration of globoside is available, one amino acid substitution of Arg296Gln sufficiently confers human protein with FS activity.

### Forssman glycolipid synthase activity of mouse *ABO* gene-encoded *cis*-AB transferase

As mentioned above, mouse *ABO* gene-encoded *cis*-AB transferase contains the GlyGlyAla tripeptide sequence, which is also found in the majority of *GBGT1* genes. Therefore, we examined FS activity of mouse *cis*-AB transferase construct (M_ABO-AB), using COS1(B3GALNT1) cells with a higher globoside substrate concentration. In addition to mouse *GBGT1* construct (M_GBGT1) as a positive control, we also analyzed human A, B, and *cis*-AB transferase constructs (H_ABO-A, H_ABO-B, and H_ABO-AB, respectively). Results of immunostaining are shown in Panel (I) of Table 1. We could detect cell surface expression of Forssman antigen on some of COS1(B3GALNT1) cells transfected with M_ABO-AB expression construct. Only the mouse *cis*-AB transferase with the GlyGlyAla tripeptide sequence, but not the human *cis*-AB transferase with the LeuGlyAla, exhibited FS activity equivalent of mouse *GBGT1* gene-encoded FS.

### Abolished FS activity of mouse *GBGT1* gene-encoded FS and mouse *ABO* gene-encoded *cis*-AB transferase by substituting GlyGlyAla tripeptide with LeuGlyGly or MetGlyAla, and acquired weak FS activity of human A transferase by substituting LeuGlyGly with GlyGlyAla

**Figure 3:**
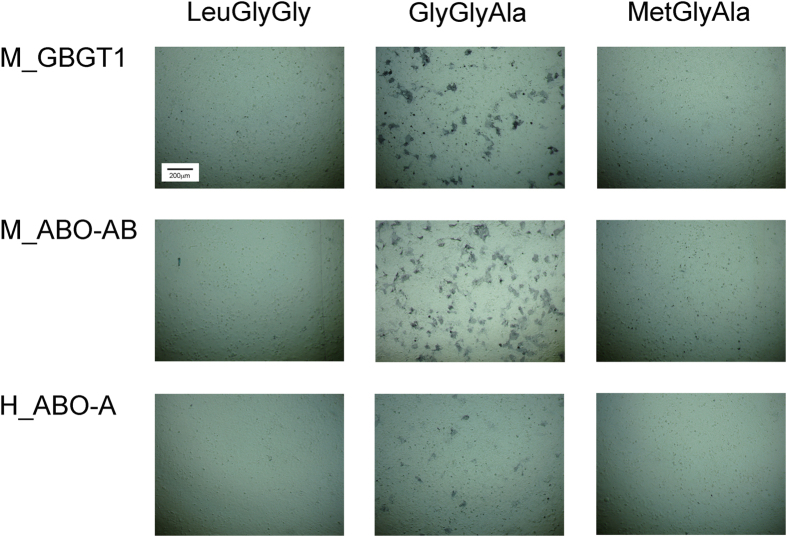
Forssman antigen expression of COS1(B3GALNT1) cells transfected with the original and amino acid substitution constructs of mouse *GBGT1* gene, mouse *ABO-AB* gene, and human *ABO-A* gene. Results of anti-Forssman antibody immunostaining of COS1(B3GALNT1) cells after DNA transfection are shown. Cells were imaged in color using a Leica DMIL LED microscope equipped with D-LUX3 camera. All images were taken at the same magnification using a 10x/0.25 PH1 objective. Figures were created using Adobe Photoshop software. The eukaryotic expression constructs prepared in the pSG5 plasmid vector were used. The results are: mouse original *GBGT1* gene (M_GBGT1) having GlyGlyAla tripeptide and its *in vitro* mutagenized amino acid substitution constructs with either LeuGlyGly or MetGlyAla tripeptide in the top row, mouse original *ABO-AB* gene (M_ABO-AB) with GlyGlyAla tripeptide and its substitution constructs having LeuGlyGly or MetGlyAla in the middle row, and human *A* allele of *ABO* gene (H_ABO-A) with LeuGlyGly tripeptide and its substitution constructs having GlyGlyAla or MetGlyAla tripeptide in the bottom row. Under the experimental conditions used, cells became confluent and formed a monolayer. Strong positive staining with anti-Forssman antibody was observed by DNA transfection of original M_GBGT1 and M_ABO-AB expression constructs. Additionally, COS1(B3GALNT1) cells transfected with H_ABO-A(GGA), the H_ABO-A construct with GlyGlyAla tripeptide sequence, exhibited some positive staining. It should be noted that not all cells were stained positive by DNA transfection as opposed to the viral infection-FACS sorting results shown in Fig. 2. Bluish background color was due to DAB and nickel ions in color development solution.

We next prepared the following amino acid substitution constructs of *ABO* gene-encoded transferases by *in vitro* mutagenesis: M_ABO-AB(LGG), M_ABO-AB(MGA), and H_ABO-A(GGA). Similarly, we also prepared the substitution constructs of mouse *GBGT1* gene-encoded FS: M_GBGT1(LGG) and M_GBGT1(MGA). The LeuGlyGly and MetGlyAla tripeptide sequences are found in A and B transferases from various species. Together with H_ABO-A, H_ABO-A(MGA), M_ABO-AB, and M_GBGT1, those constructs were transfected to COS1(B3GALNT1) cells. Results of immunostaining after DNA transfection are shown in Panel (II) of Table 1. Several photographs of the immunostained cells transfected with different constructs are shown for comparison in Fig. 3.

The substitution of GlyGlyAla sequence of mouse *cis*-AB transferase by either A transferase-specific LeuGlyGly or B transferase-specific MetGlyAla eliminated FS activity, suggesting that the GlyGlyAla tripeptide sequence is important for FS activity of mouse *cis*-AB transferase. The GlyGlyAla substitution of mouse FS by LeuGlyGly or MetGlyAla also abolished FS activity, indicating that this sequence is also important for FS activity of *GBGT1* gene-encoded FSs. On the other hand, the substitution of LeuGlyGly of human A transferase by FS-specific GlyGlyAla conferred some FS activity although the activity was less than mouse FS or *cis*-AB transferase. In order to obtain more quantitative data, we also performed immunocytometry of the cells transfected with the selected constructs. After DNA transfection, cells were resuspended and immunostained. In combination with anti-Forssman antibody, Alexa Fluor 488-conjugated secondary antibody was used for fluorescence detection. Forssman antigen-positive cell percentages and Median Fluorescence Intensity (MFI) values of positive cell populations were determined. Results are shown in Table 2, confirming that some of the COS1(B3GALNT1) cells transfected with the H_ABO-A(GGA) construct, but not with the H_ABO-A construct, expressed Forssman antigen. The positive cell percentages, as well as the MFI values, of the H_ABO-A(GGA) construct were much lower than those of M_GBGT1 or M_ABO-AB. We, therefore, concluded that the GlyGlyAla tripeptide sequence alone is not sufficient for human A transferase to acquire a full FS activity. Some other determinants must be necessary.

## Discussion

Immunochemical determination of glycolipid fractions of RBCs from individuals exhibiting A_pae_ phenotype led to the discovery of a novel blood group system of FORS^16^. However, our results presented here suggest that the boundary between ABO and Forssman systems may not be as strictly delineated as previously thought.

Both *ABO* and *GBGT1* genes are members of evolutionary related α1,3-Gal(NAc) transferase family of genes, deriving from the same ancestral gene^29^. *ABO* genes seem to have appeared during/after the separation of amphibians from fish. *FUT1/FUT2/Sec* genes encoding α1,2-fucosyltransferases that catalyze the last biosynthetic pathway of H substance, the acceptor substrate of A/B transferases, also seem to have emerged in amphibians. Accordingly, it is more logical to hypothesize that *ABO* gene-encoded A/B transferases have acquired the capability of utilizing H substance, a fucosylated acceptor substrate, than to assume that *GBGT1* gene-encoded FSs have lost the ability to utilize fucosylated acceptor substrates. Even with this acquisition, however, *ABO* gene-encoded transferases may still retain some ability to utilize unfucosylated substrates if certain conditions are met. Obviously, the GlyGlyAla tripeptide sequence, which allows *ABO* gene-encoded glycosyltransferases to catalyze the biosynthesis of the FORS1 antigen, is such an example. The evolutionary link between *ABO* and *GBGT1* genes and the structural similarity between A/B transferases and FSs make distinct blood group systems of ABO and FORS closely connected one another.

We have previously shown that two missense mutations resulting in Gly230Ser and Gln296Arg substitutions are responsible for inactivity of human *GBGT1* gene-encoded FS protein^27^. Re-examination of those inactivating mutations using COS1(B3GALNT1) cells has revealed that Arg296Gln substitution alone might bestow human *GBGT1* gene-encoded protein with FS activity strong enough to present what is called A_pae_ phenotype under the condition where sufficient globoside concentration was provided by the forced expression of β1,3-*N*-acetyl-galactosaminyltransferase 1. Actually, an augmented FS activity by *B3GALNT1* gene expression was previously reported by Svensson and colleagues, who observed a striking increase in Forssman antigen-positive cells after co-transfecting a hematopoietic cell line with *B3GALNT1* gene construct and *GBGT1* gene construct with Arg296Gln substitution^16^. Obviously, enhanced sensitivity of FS activity detection is not unique to COS1(B3GALNT1) cells. Extrapolating from this observation, deficiency in human *GBGT1* gene-encoded FS protein caused by Gln296Arg substitution may also be overcome by currently unknown differential physiological/pathological conditions.

As mentioned earlier in the Introduction section, Forssman antigen has been detected in human cancer cells and tissues^17^,^18^,^19^,^20^,^21^,^22^,^23^. In addition to immunohistochemistry and immunocytometry, antigens reactive to anti-Forssman antibodies have also been detected by immunostaining of glycolipids separated through thin-layer chromatography^17^,^23^,^31^. One may question the specificity of anti-Forssman antibodies used in those experiments because polyclonal antibodies were used in many of the studies. However, in certain cases of human cancer Forssman glycolipid was demonstrated to exist by chemical determination of the antigenic structures^18^. It should be noted that in addition to regular pentasaccharide structure, Forssman antigen with unusual core structures, such as ceramide trisaccharide and a compound longer than a pentasaccharide ceramide, have also been detected in some tumors^23^,^32^. Similarly, Forssman antigens carried on glycoproteins seem to exist^33^,^34^,^35^.

The allele frequency of single nucleotide polymorphism (SNP) (rs375748588) responsible for the A_pae_ phenotype is very low: 0.000008 in Exome Aggregation Consortium - ExAC Browser and 0.00008 in NHLBI Grand Opportunity Exome Sequencing Project (GO-ESP) database. Because this trait is dominant, the phenotype frequency of A_pae_ may be of a similar value. Accordingly, this low allele frequency does not explain more frequent observation of Forssman antigen expression in cancer. COSMIC (Catalogue of Somatic Mutations in Cancer) database lists 60 mutations found in *GBGT1* gene in various cancers, but none of them are missense mutations at codon 230 or 296. No SNPs have been found of the second nucleotide (T) of the triplet encoding Leu (CTG) or Met (ATG) at codon 266 of the *ABO* gene. Therefore, individuals with GlyGlyAla may be very rare. From regular A transferase with LeuGlyGly or B transferase with MetGlyAla, at least 3 or 2 nucleotide substitutions are necessary to change to GlyGlyAla, respectively, and actually, no mutations at the tripeptide sequence resulting in LeuGlyGly266–268GlyGlyAla or MetGlyAla266–268GlyGlyAla substitution have been reported of *ABO* gene in cancer, either. In summary, missense mutations at the tripeptide sequence are unlikely to explain Forssman antigen expression in cancer.

However, there are other potential mechanisms that remain to be examined. These include over-expression of *GBGT1* gene mRNA and FS protein and increased stability of mRNA and protein. Splicing variations and post-translational protein modifications may also change the specificity and activity of FS protein. Even if no changes are observed in the structure and quantity of FS protein, extraordinarily higher concentrations of UDP-GalNAc and/or globoside substrates than threshold levels in the Golgi apparatus may also achieve the FS function. The finding that increased availability of globoside complemented the Gly230Ser deficiency supports this argument. Additionally, certain tumors were shown to contain higher concentrations of UDP-GalNAc and UDP-GlcNAc^36^. An increased level of intracellular UDP-GlcNAc was shown to activate O-GlcNAc transferase (OGT) and lead to enhanced O-GlcNAcylation of target proteins^37^. Therefore, a similar mechanism based on high donor nucleotide-sugar concentration may also influence the Forssman antigen expression. Forssman-positive cells tend to appear after DNA transfection on dish edges where cells are more crowded, suggesting that cell density may affect the antigen expression or antibody binding. Our results have proven that Forssman antigen might also be produced by other glycosyltransferases than FS. The observation that mouse *ABO* gene encodes a protein with FS activity warrants further exploration of the possibility that blood group *ABO* gene-encoded transferases may be involved in the biosynthesis of Forssman antigen in human cancer by yet-to-be defined molecular mechanisms.

## Methods

### Retrieval of *ABO* and *GBGT1* gene/protein sequences and comparison of amino acid sequences of human A/B/*cis*-AB transferases, mouse *cis*-AB transferase, mouse Forssman glycolipid synthase (FS), and human FS proteins

The Mega file (Mouse_*GBGT1*_orthologues.med.txt) that aligned amino acid sequences of *GBGT1* genes from a variety of species was downloaded from Ensembl database. The amino acid sequences of human A, B, and *cis*-AB transferases encoded by *A101, B101*, and *cis*-*AB01* alleles at the *ABO* genetic locus were modified from the protein sequence of Ensembl ABO-202 transcript (ENST00000611156) by adding 3 nucleotides at the exon-intron splicing junction, which were missing in the annotated sequence, and then introducing allele-specific amino acid differences that were deposited at the dbRBC - BGMUT - System - ABO – NCBI database (http://www.ncbi.nlm.nih.gov/gv/rbc/xslcgi.fcgi?cmd=bgmut/systems_info&system=abo). The amino acid sequences of mouse *cis*-AB transferase, as well as human and mouse FS proteins, were retrieved from Ensembl, and directly used for sequence alignment, using ClustralW software of MEGA5 that was available online^38^.

### Preparation of COS1(B3GALNT1) cells by retroviral introduction of human *B3GALNT1* gene

African green monkey kidney COS1 cells were originally obtained from American Type Culture Collection (ATCC), and have been maintained in the laboratory. In order to improve the sensitivity of detection of FS activity, we created COS1(B3GALNT1) cells that produce increased number of globoside, the acceptor substrate of FS. For this purpose, we first prepared retroviral construct pMigR1b-B3GALNT1 by inserting human *B3GALNT1* (β1,3-*N*-acetyl-galactosaminyltransferase 1) gene cDNA into pMigR1b (mTagBFP) vector at the cloning site preceding the internal ribosome entry sequence (IRES) element followed by blue fluorescent protein (BFP) gene cDNA. This vector construct was previously used to demonstrate the feasibility of preparing custom-made murine cells exhibiting specific glycolipid profiles by modular expression of extraneously introduced glycosyltransferase genes^39^. The pMigR1b-B3GALNT1 construct was transfected into packaging Phoenix-AMPHO cells. Then Phoenix-AMPHO medium containing the viral particles was recovered, filtered, and added to COS1 cells. The cells infected were allowed to grow to confluence, detached/suspended with trypsin/EDTA, and cells expressing coupled BFP were FACS-sorted. Those selected cells, COS1(B3GALNT1), were later used as hosts of subsequent viral infection and recipients of DNA transfection.

### Preparation and infection of viral vectors of human *GBGT1* gene and its derivatives

The expression constructs prepared in pSG5 (Stratagene) plasmid vector of human *GBGT1* (H_GBGT1) and human *GBGT1* with Ser230Gly, Arg296Gln, or both substitutions (H_GBGT1-S230G, H_GBGT1-R296Q, and H_GBGT1-S230G&R296Q, respectively), were previously described^27^. Those inserts were subcloned into pMigR1 (EGFP) viral vector having coupled enhanced green fluorescent protein (EGFP) gene cDNA, and the constructs were then transfected into Phoenix-AMPHO cells. The viral particles were produced and infected to COS1 and COS1(B3GALNT1) cells using the same strategy explained above. EGFP positive cells were selected of COS1 cells by FACS sorting. Double positive cells with EGFP and mTagBFP were selected of COS1(B3GALNT1) cells. The isolated populations were amplified and then seeded onto glass coverslips for Forssman antigen immunodetection.

### Construction of *in vitro* mutagenized plasmid expression constructs

The constructs were prepared in pSG5. The original expression constructs of human A and B transferases (H_ABO-A and H_ABO-B), mouse *cis*-AB transferase (M_ABO-AB), and mouse FS (M_GBGT1), as well as human A transferase construct with Leu266Met and Gly268Ala substitutions (H_ABO-A(MGA)), were previously described^26^,^27^,^28^. The *in vitro* mutagenized constructs were prepared through a two-round PCR amplification using specific primers with nucleotide substitutions. The names and nucleotide sequences of primers, as well as detailed PCR protocols, can be found in Supplemental Information. The constructs without any additional unintended non-synonymous mutations were used in the following experiments.

### DNA transfection

COS1(B3GALNT1) cells were used as recipients of DNA transfection. Lipofectamine 3000 reagents were used, following the manufacturer’s protocol (Life Technologies). For immunological detection of Forssman antigen by peroxidase staining procedure, two sets of expression constructs were DNA transfected: **Set 1.** Human A, B, and *cis*-AB transferase constructs (H_ABO-A, H_ABO-B, and H_ABO-AB, respectively), mouse *cis*-AB transferase construct (M_ABO-AB), and mouse *GBGT1* construct (M_GBGT1). **Set 2.** Mouse *cis*-AB transferase construct (M_ABO-AB), mouse *cis*-AB transferase constructs with either GlyGlyAla245-247LeuGlyGly or GlyGlyAla245-247MetGlyAla substitution, M_ABO-AB(LGG) and M_ABO-AB(MGA), respectively, mouse *GBGT1* construct (M_GBGT1), mouse *GBGT1* constructs with either GlyGlyAla261-263LeuGlyGly or GlyGlyAla261-263MetGlyAla substitution, M_GBGT1(LGG) and M_GBGT1(MGA), respectively, and human A transferase construct (H_ABO-A), and human A transferase constructs with LeuGlyGly266-268GlyGlyAla or LeuGlyGly266-268MetGlyAla substitution, H_ABO-A(GGA) and H_ABO-A(MGA), respectively. In order to facilitate normalization of DNA transfection efficiency, a fixed amount of pEGFP construct expressing the enhanced GFP was co-transfected. GFP-positive cells were counted under the fluorescence microscope 3 days after DNA transfection just before cell fixation with 4% paraformaldehyde. The experiments were repeated 4 times for Set 2. For immunological detection of Forssman antigen by cytometry, only the selected expression constructs were DNA transfected: **Set 3.** H_ABO-A, H_ABO-A(GGA), M_ABO-AB, M_ABO-AB(LGG), M_GBGT1, and M_GBGT1(LGG).

### Immunological detection of Forssman antigen

Immunostaining was performed using clone FOM-1 anti-Forssman Rat IgM monoclonal antibody from BMA Biomedicals. For immunoperoxidase staining procedure, cells fixed on culture plates were first incubated with the primary antibody, then with a biotinylated Goat Anti-Rat IgG + IgM(H + L) secondary antibody (Jackson ImmunoResearch Laboratories), followed by the treatment with Vectastain ABC reagents (Vector Laboratories). DAB reagents were used for peroxidase-mediated color development. Positive cell numbers were counted under the microscope and then normalized using cell numbers positive with co-transfected GFP. The values in percentage were then obtained, calculating the results of transfection with mouse FS construct (M_GBGT1) and a negative control to be 100 and 0, respectively. Pictures of the cells were also taken. For cytometry, DNA-transfected cells were detached from culture plates with EDTA, washed, and subjected to immunostaining, first with the anti-Forssman antibody and then with Alexa Fluor 488-conjugated Goat Anti-Rat IgM(μ Chain Specific) secondary antibody (Thermo Scientifics). BD LSRFortessa Analyzer (BD Biosciences) was used to determine Forssman antigen-expressing cell percentages and the B530-A Median Fluorescence Intensity (MFI) values of those expressing cell populations.

## Additional Information

**How to cite this article**: Yamamoto, M. *et al*. Crosstalk between ABO and Forssman (FORS) blood group systems: FORS1 antigen synthesis by *ABO* gene-encoded glycosyltransferases. *Sci. Rep.* **7**, 41632; doi: 10.1038/srep41632 (2017).

**Publisher's note:** Springer Nature remains neutral with regard to jurisdictional claims in published maps and institutional affiliations.

## Supplementary Material

Supplemental Table 1

Supplemental Information

## Figures and Tables

**Table 1:** Forssman antigen expression after DNA transfection of expression constructs into COS1 (B3GALNT1) cells analyzed by immunoperoxidase staining procedure.

Gene Name	Tripeptide Sequence	Adjusted Forssman Antigen-Positive Cell %				
		Exp. 1	Exp. 2	Exp. 3	Exp. 4	Exp. 5
**(I)**						
M_GBGT1	GlyGlyAla	**100**				
H_ABO-A	LeuGlyGly	0				
H_ABO-B	MetGlyAla	0				
H_ABO-AB	LeuGlyAla	0				
M_ABO-AB	GlyGlyAla	100				
**(II)**						
M_GBGT1	GlyGlyAla		**100**	**100**	**100**	**100**
	LeuGlyGly		0	0	0	0
	MetGlyAla		0	0	0	0
M_ABO-AB	GlyGlyAla		150	69	105	76
	LeuGlyGly		0	0	0	0
	MetGlyAla		0	0	0	0
H_ABO-A	LeuGlyGly		0	0	0	0
	MetGlyAla		0	0	0	0
	GlyGlyAla		12	8	19	13

Two sets of experiments were performed, and the results are shown in Panels (I) and (II). The original construct names are listed in the leftmost column. The tripeptide sequences corresponding to codons 266–268 of human A/B/*cis*-AB transferases are shown in the second column from the left. The results of immunostaining with FOM-1 anti-Forssman antibody are shown in the 5 rightmost columns. The numbers of cells positive with enhanced GFP, whose expression vector was co-transfected, were used to normalize Forssman antigen positivity. The values are shown in percentage of the expression observed of the original mouse FS (M_GBGT1) construct to be 100.

**Table 2:** Forssman antigen expression after DNA transfection of expression constructs into COS1 (B3GALNT1) cells analyzed by immunocytometry.

Gene Name	Tripeptide Sequence	Exp. 1		Exp. 2		Exp. 3	
		Pos Cell %	MFI	Pos Cell %	MFI	Pos Cell %	MFI
H_ABO-A	LeuGlyGly			0.0		0.0	
	GlyGlyAla	1.8	15855	1.8	18325	0.8	16023
M_ABO-AB	LeuGlyGly	0.0		0.0		0.0	
	GlyGlyAla	13.8	48490	9.3	28912	9.6	26463
M_GBGT1	LeuGlyGly			0.0		0.0	
	GlyGlyAla	22.8	81893	19.1	36711	25.6	43306

The original construct names are listed in the leftmost column. The tripeptide sequences corresponding to codons 266–268 of human A/B/*cis*-AB transferases are shown in the second column from the left. The results of immunostaining with FOM-1 anti-Forssman antibody in 3 separate experiments are shown in the 6 rightmost columns. The Forssman antigen-positive cell percentages, as well as the MFI values of those antigen-positive cell populations, are indicated.
