# Supplementary material for: Crosstalk between ABO and Forssman (FORS) blood group systems: FORS1 antigen synthesis by ABO gene-encoded glycosyltransferases
Source: Sci Rep. 2017 Jan 30;7:41632. doi: 10.1038/srep41632 (PMC5278553; doi:10.1038/srep41632)
Supplement: Supplemental Table 1 [file srep41632-s1.doc]

**Title:**

Crosstalk between ABO and Forssman (FORS) blood group systems: FORS1 antigen synthesis by *ABO* gene-encoded glycosyltransferases

**Authors:**

Miyako Yamamoto, Emili Cid, and Fumiichiro Yamamoto

| **Supplemental Table 1. The GBGT1 gene orthologues** | |  |  |  |
| --- | --- | --- | --- | --- |
|  |  |  |  |  |
| **Species** | **Ensembl gene identifier** | **Ensembl protein identifier** | **Tripeptide** | **Potential inactivation cause** |
| **Amazon molly (Poecilia formosa)** | ENSPFOG00000016123 | ENSPFOP00000016238 | **TAA** |  |
| **Amazon molly (Poecilia formosa)** | ENSPFOG00000017818 | ENSPFOP00000017906 | **CGG** |  |
| Armadillo (Dasypus novemcinctus) | ENSDNOG00000034893 | ENSDNOP00000031947 | GGA |  |
| Bushbaby (Otolemur garnettii) | ENSOGAG00000029130 | ENSOGAP00000015742 | GAA |  |
| Cat (Felis catus) | ENSFCAG00000024387 | ENSFCAP00000016899 | GGA |  |
| **Cave fish (Astyanax mexicanus)** | ENSAMXG00000025723 | ENSAMXP00000026428 | **TSE** |  |
| Chicken (Gallus gallus) | ENSGALG00000003340 | ENSGALP00000005275 | GGA |  |
| Chimpanzee (Pan troglodytes) | ENSPTRG00000021510 | ENSPTRP00000036809 | GGA |  |
| Chinese softshell turtle (Pelodiscus sinensis) | ENSPSIG00000014027 | ENSPSIP00000015723 | GGA |  |
| **Cod (Gadus morhua)** | ENSGMOG00000012375 | ENSGMOP00000013247 | **SAA** |  |
| Coelacanth (Latimeria chalumnae) | ENSLACG00000000483 | ENSLACP00000000544 | GGA |  |
| **Cow (Bos taurus)** | ENSBTAG00000030319 | ENSBTAP00000040428 | **GRA** | 11 aa deletion after the triplet |
| Dog (Canis lupus familiaris) | ENSCAFG00000019864 | ENSCAFP00000029424 | GGA |  |
| Dolphin (Tursiops truncatus) | ENSTTRG00000014438 | ENSTTRP00000013692 | GGA |  |
| Duck (Anas platyrhynchos) | ENSAPLG00000004843 | ENSAPLP00000004366 | GGA |  |
| Elephant (Loxodonta africana) | ENSLAFG00000005668 | ENSLAFP00000004756 | GGA |  |
| Ferret (Mustela putorius furo) | ENSMPUG00000012426 | ENSMPUP00000012332 | GGA |  |
| Flycatcher (Ficedula albicollis) | ENSFALG00000001494 | ENSFALP00000001555 | GGA |  |
| Gorilla (Gorilla gorilla gorilla) | ENSGGOG00000003448 | ENSGGOP00000003390 | GGA |  |
| Guinea Pig (Cavia porcellus) | ENSCPOG00000004874 | ENSCPOP00000004388 | GGA |  |
| Hedgehog (Erinaceus europaeus) | ENSEEUG00000014255 | ENSEEUP00000013001 | GGA |  |
| Horse (Equus caballus) | ENSECAG00000012442 | ENSECAP00000010230 | GGA |  |
| Human (Homo sapiens) | ENSG00000148288 | ENSP00000361110 | GGA |  |
| Hyrax (Procavia capensis) | ENSPCAG00000015568 | ENSPCAP00000014521 | GGA |  |
| Kangaroo rat (Dipodomys ordii) | ENSDORG00000011384 | ENSDORP00000010699 | GGA |  |
| Lesser hedgehog tenrec (Echinops telfairi) | ENSETEG00000011084 | ENSETEP00000008989 | GGA |  |
| **Macaque (Macaca mulatta)** | ENSMMUG00000015277 | ENSMMUP00000020033 | **GGK** | Lysine may abolish the activity. |
| **Medaka (Oryzias latipes)** | ENSORLG00000010140 | ENSORLP00000012717 | **TAA** |  |
| Megabat (Pteropus vampyrus) | ENSPVAG00000001029 | ENSPVAP00000000973 | GGA |  |
| Mouse (Mus musculus) | ENSMUSG00000026829 | ENSMUSP00000127071 | GGA |  |
| Mouse Lemur (Microcebus murinus) | ENSMICG00000010686 | ENSMICP00000009738 | GGA |  |
| **Olive baboon (Papio anubis)** | ENSPANG00000018174 | ENSPANP00000006276 | **GGK** | Lysine may abolish the activity. |
| Opossum (Monodelphis domestica) | ENSMODG00000012666 | ENSMODP00000015845 | GGA |  |
| Orangutan (Pongo abelii) | ENSPPYG00000019724 | ENSPPYP00000022121 | GGA |  |
| **Platyfish (Xiphophorus maculatus)** | ENSXMAG00000012659 | ENSXMAP00000012692 | **CGG** |  |
| **Platyfish (Xiphophorus maculatus)** | ENSXMAG00000014479 | ENSXMAP00000014536 | **TAA** |  |
| Platypus (Ornithorhynchus anatinus) | ENSOANG00000008304 | ENSOANP00000013200 | GGA |  |
| Sheep (Ovis aries) | ENSOARG00000005261 | ENSOARP00000005644 | GGA |  |
| Sloth (Choloepus hoffmanni) | ENSCHOG00000002651 | ENSCHOP00000002348 | GGA |  |
| **Spotted gar (Lepisosteus oculatus)** | ENSLOCG00000000065 | ENSLOCP00000000075 | **---** |  |
| **Spotted gar (Lepisosteus oculatus)** | ENSLOCG00000000741 | ENSLOCP00000000822 | **QAA** |  |
| Spotted gar (Lepisosteus oculatus) | ENSLOCG00000004182 | ENSLOCP00000004989 | GGA |  |
| **Stickleback (Gasterosteus aculeatus)** | ENSGACG00000000537 | ENSGACP00000000689 | **TAA** |  |
| **Stickleback (Gasterosteus aculeatus)** | ENSGACG00000014813 | ENSGACP00000019565 | **CGG** |  |
| **Stickleback (Gasterosteus aculeatus)** | ENSGACG00000016064 | ENSGACP00000021194 | **TAA** |  |
| Tasmanian devil (Sarcophilus harrisii) | ENSSHAG00000008459 | ENSSHAP00000009774 | GGA |  |
| **Tilapia (Oreochromis niloticus)** | ENSONIG00000009223 | ENSONIP00000011602 | **CGG** |  |
| Tree Shrew (Tupaia belangeri) | ENSTBEG00000010196 | ENSTBEP00000008812 | GGA |  |
| Turkey (Meleagris gallopavo) | ENSMGAG00000006307 | ENSMGAP00000006329 | GGA |  |
| **Vervet-AGM (Chlorocebus sabaeus)** | ENSCSAG00000015155 | ENSCSAP00000011245 | **GGK** | Lysine may abolish the activity. |
| Zebra Finch (Taeniopygia guttata) | ENSTGUG00000005430 | ENSTGUP00000005586 | GGA |  |
| **Zebrafish (Danio rerio)** | ENSDARG00000005257 | ENSDARP00000018621 | **TAA** |  |
| **Zebrafish (Danio rerio)** | ENSDARG00000011283 | ENSDARP00000015549 | **TAA** |  |
| **Zebrafish (Danio rerio)** | ENSDARG00000019207 | ENSDARP00000010651 | **CGA** |  |
| **Zebrafish (Danio rerio)** | ENSDARG00000025275 | ENSDARP00000018900 | **TAA** |  |
| Zebrafish (Danio rerio) | ENSDARG00000035555 | ENSDARP00000051540 | GGA |  |
| Zebrafish (Danio rerio) | ENSDARG00000068503 | ENSDARP00000120652 | GGA |  |
| **Zebrafish (Danio rerio)** | ENSDARG00000091936 | ENSDARP00000112590 | **---** |  |
| Zebrafish (Danio rerio) | ENSDARG00000091944 | ENSDARP00000139562 | GGA |  |
| Zebrafish (Danio rerio) | ENSDARG00000091969 | ENSDARP00000122748 | GGA |  |
| Zebrafish (Danio rerio) | ENSDARG00000092718 | ENSDARP00000114822 | GGA |  |
|  |  |  |  |  |
|  |  |  |  |  |
| **Species without orthologues** |  |  |  |  |
| **23 species shown below don't have any GBGT1 orthologues.** | |  |  |  |
|  |  |  |  |  |
| Ciona intestinalis |  |  |  |  |
| Ciona savignyi |  |  |  |  |
| Alpaca (Vicugna pacos) |  |  |  |  |
| Anole lizard (Anolis carolinensis) |  |  |  |  |
| Caenorhabditis elegans (Caenorhabditis elegans) |  |  |  |  |
| Fruitfly (Drosophila melanogaster) |  |  |  |  |
| Fugu (Takifugu rubripes) |  |  |  |  |
| Gibbon (Nomascus leucogenys) |  |  |  |  |
| Lamprey (Petromyzon marinus) |  |  |  |  |
| Marmoset (Callithrix jacchus) |  |  |  |  |
| Microbat (Myotis lucifugus) |  |  |  |  |
| Panda (Ailuropoda melanoleuca) |  |  |  |  |
| Pig (Sus scrofa) |  |  |  |  |
| Pika (Ochotona princeps) |  |  |  |  |
| Rabbit (Oryctolagus cuniculus) |  |  |  |  |
| Rat (Rattus norvegicus) |  |  |  |  |
| Saccharomyces cerevisiae (Saccharomyces cerevisiae) |  |  |  |  |
| Shrew (Sorex araneus) |  |  |  |  |
| Squirrel (Ictidomys tridecemlineatus) |  |  |  |  |
| Tarsier (Tarsius syrichta) |  |  |  |  |
| Tetraodon (Tetraodon nigroviridis) |  |  |  |  |
| Wallaby (Macropus eugenii) |  |  |  |  |
| Xenopus (Xenopus tropicalis) |  |  |  |  |
|  |  |  |  |  |
|  |  |  |  |  |
| **Note** |  |  |  |  |
| Species containing the tripeptide sequence other than GGA are shown in bold type. | | |  |  |
| Among them fish species are shown in red, whereas mammalian species are shown in blue. | | |  |  |
