# Supplementary material for: Crosstalk between ABO and Forssman (FORS) blood group systems: FORS1 antigen synthesis by ABO gene-encoded glycosyltransferases
Source: Sci Rep. 2017 Jan 30;7:41632. doi: 10.1038/srep41632 (PMC5278553; doi:10.1038/srep41632)
Supplement: Supplemental Information [file srep41632-s2.pdf]

Title:

Crosstalk between ABO and Forssman (FORS) blood group systems: FORS1 antigen synthesis by *ABO* gene-encoded glycosyltransferases

Authors:

Miyako Yamamoto, Emili Cid, and Fumiichiro Yamamoto

Supplemental Information

Experimental Protocol

Construction of *in vitro* mutagenized amino acid substitution constructs

The N-ter primers contain an artificial *EcoRI* restriction cleavage site, and the C-ter primers contain a *BamHI* site for easier cloning with the *EcoRI-BamHI* fragment of the expression vector pSG-5. Using the diluted *XbaI*-digested plasmid DNA from the original constructs as PCR templates, two-step PCR reactions were performed using AccuPrimeÊ Pfx SuperMix. The N-ter and C-ter DNA fragments containing nucleotide substitutions at their ends were initially amplified separately. Then, the 2 reactions were mixed, and an additional PCR cycle was performed to amplify the full-length DNA fragments containing the mutations. The reaction products were purified using the PureLinkÊ Quick PCR Purification Kit, and digested with *EcoRI* and *BamHI*. After purification, *EcoRI-BamHI* DNA fragments were ligated to the dephosphorylated vector DNA digested with *EcoRI* and *BamHI*. After DNA transformation of frozen competent *Echerchia coli* bacteria, several transformant clones were selected. Plasmid DNA was prepared from those clones, using ChargeSwitchÊ -Pro Plasmid Miniprep kit. DNA sequencing was performed, and the clones with the intended amino acid substitutions without any additional non-synonymous mutations were selected and used for DNA transfection experiments. All reagents and kits used for the plasmid constructions were purchased from Invitrogen-Life Technologies. Custom DNA sequencing was performed at GATC Biotech.

PCR primers

The primers used are as follows.

```
H_ABO-A(N-ter_F):    5'-CCCGGAATTCCATGGCCGAGGTGTTGCGGA
H_ABO-A(C-ter_R):    5'-CCCGGGATCCGCTCACGGGTTCCGGACCGC
M_ABO-AB(N-ter_F):   5'-CCGGAATTCTGACATGAATCTCAGAGGAAG
M_ABO-AB(C-ter_R):   5'-CCCGGATCCAATAGGAATTTAGCTATTAGT
M_GBGT1(N-ter_F):    5'-CCGGAATTCTGCACCCAGTGATGACCCGCC
M_GBGT1(C-ter_R):    5'-CCCGGATCCTTAGCCCGGATGTTAGGTCCT
H_ABO-A(GGA_F):      5'-TTCTACTACGGGGGGGCGTTCTTCGG
H_ABO-A(GGA_R):      5'-AGAACGCCCCCCCGTAGTAGAAATCG
M_ABO-AB(LGG_F):     5'-TTTACTACCTAGGAGGCTTCTTTGGG
M_ABO-AB(LGG_R):     5'-AGAAGCCTCCTAGGTAGTAAAAGTCA
M_ABO-AB(MGA_F):     5'-TCTATTATATGGGGGCACTCTTTGGGGG
M_ABO-AB(MGA_R):     5'-CCCCCAAAGAGTGCCCCCATATAATAGA
M_GBGT1(LGG_F):      5'-TCTATTATCTTGGGGGACTCTTTGGGGG
M_GBGT1(LGG_R):      5'-CCCCCAAAGAGTCCCCCAAGATAATAGA
M_GBGT1(MGA_F):      5'-TACTACATGGGAGCCTTCTTTGGGGGGTC
M_GBGT1(MGA_R):      5'-GACCCCCCAAAGAAGGCTCCCATGTAGTAA
```

PCR template-primer combinations

Five *in vitro* mutagenized constructs were prepared.

1. H_ABO-A(GGA)

PCR 1-1
Template H_ABO-A/XbaI dil
Primers H_ABO-A(N-ter_F) & H_ABO-A(GGA_R)
PCR 1-2
Template H_ABO-A/XbaI dil
Primers H_ABO-A(GGA_F) & H_ABO-A(C-ter_R)

PCR2
Mix PCR 1-1 & 1-2

2. M_ABO-AB(LGG)

PCR 1-1
Template M_ABO-AB/XbaI dil
Primers M_ABO-AB(N-ter_F) & M_ABO-AB(LGG_R)

PCR 1-2
Template M_ABO-AB/XbaI dil
Primers M_ABO-AB(LGG_F) & M_ABO-AB(C-ter_R)

PCR2
Mix PCR 1-1 & 1-2

3. M_ABO-AB(MGA)

PCR 1-1
Template M_ABO-AB/XbaI dil
Primers M_ABO-AB(N-ter_F) & M_ABO-AB(MGA_R)

PCR 1-2
Template M_ABO-AB/XbaI dil
Primers M_ABO-AB(MGA_F) & M_ABO-AB(C-ter_R)

PCR2
Mix PCR 1-1 & 1-2

4. M_GBGT1(LGG)

PCR 1-1
Template M_GBGT1/XbaI dil
Primers M_GBGT1(N-ter_F) & M_GBGT1(LGG_R)

PCR 1-2
Template M_GBGT1/XbaI dil
Primers M_GBGT1(LGG_F) & M_GBGT1(C-ter_R)

PCR2

Mix PCR 1-1 & 1-2

5. M_GBGT1(MGA)

PCR 1-1
Template M_GBGT1/XbaI dil
Primers M_GBGT1(N-ter_F) & M_GBGT1(MGA_R)

PCR 1-2
Template M_GBGT1/XbaI dil
Primers M_GBGT1(MGA_F) & M_GBGT1(C-ter_R)

PCR2
Mix PCR 1-1 & 1-2

PCR reaction conditions

Reaction mixture

9µl	AccuPrimeÊ Pfx SuperMix
2µl	10pmol/µl of external primer
0.3µl	10pmol/µl of internal primer
0.3µl	100pg/µl of diluted *XbaI*-digested template DNA

Reaction cycles

PCR 1
2 min at 94°C, followed by 18 cycles of 30 sec at 94°C, 30 sec at 42°C, and 2 min at 68°C.

Mixing of the two reaction products

PCR 2
2 min at 94°C, 20 cycles of 30 sec at 94°C, 30 sec at 46°C, and 2 min at 68°C, followed by 10 min at 68°C
